# K-nearest neighbor algorithm for imputing missing longitudinal prenatal alcohol data

**DOI:** 10.3389/adar.2024.13449

**Published:** 2025-01-28

**Authors:** Ayesha Sania, Nicolò Pini, Morgan E. Nelson, Michael M. Myers, Lauren C. Shuffrey, Maristella Lucchini, Amy J. Elliott, Hein J. Odendaal, William P. Fifer

**Affiliations:** ^1^ Department of Psychiatry, Columbia University Irving Medical Center, New York, NY, United States; ^2^ Division of Developmental Neuroscience, New York State Psychiatric Institute, New York, NY, United States; ^3^ Research Triangle Institute, Research Triangle Park, Durham, NC, United States; ^4^ Department of Child and Adolescent Psychiatry, NYU Grossman School of Medicine, New York, NY, United States; ^5^ Center for Pediatric and Community Research, Avera Health, Sioux Falls, SD, United States; ^6^ Department of Pediatrics, University of South Dakota School of Medicine, Sioux Falls, SD, United States; ^7^ Department of Obstetrics and Gynecology, Faculty of Medicine and Health Science, Stellenbosch University, Cape Town, Western Cape, South Africa

**Keywords:** k nearest neighbor, *k-NN*, machine learning, data missingness, data imputation, prenatal alcohol data

## Abstract

**Aims:**

The objective of this study is to illustrate the application of a machine learning algorithm, K Nearest Neighbor (*k-NN*) to impute missing alcohol data in a prospective study among pregnant women.

**Methods:**

We used data from the Safe Passage study (n = 11,083). Daily alcohol consumption for the last reported drinking day and 30 days prior was recorded using the Timeline Follow back method, which generated a variable amount of missing data per participants. Of the 3.2 million person-days of observation, data were missing for 0.36 million (11.4%). Using the *k-NN* imputed values were weighted for the distances and matched for the day of the week. Since participants with no missing days were not comparable to those with missing data, segments of non-missing data from all participants were included as a reference. Validation was done after randomly deleting data for 5–15 consecutive days from the first trimester.

**Results:**

We found that data from 5 nearest neighbors (i.e., K = 5) and segments of 55 days provided imputed values with least imputation error. After deleting data segments from the first trimester data set with no missing days, there was no difference between actual and predicted values for 64% of deleted segments. For 31% of the segments, imputed data were within +/−1 drink/day of the actual. Imputation accuracy varied by study site because of the differences in the magnitude of drinking and proportion of missing data.

**Conclusion:**

*k-NN* can be used to impute missing data from longitudinal studies of alcohol during pregnancy with high accuracy.

## Introduction

Accurate assessment of timing, frequency, and quantity of prenatal alcohol exposure in longitudinal research studies is necessary for obtaining unbiased assessments of the effects on fetal and infant outcomes. Despite the recent development of several biomarkers that assess the presence of alcohol exposure during pregnancy [1], these markers have limited sensitivity in detecting the timing and amount of alcohol exposure during pregnancy [2, 3]. Thus, we often remain reliant on maternal self-report of intake. Aside from issues associated with the accuracy of self-report, there are other methodological challenges in measuring alcohol exposure in longitudinal studies [4, 5]. Recording daily intake, while providing a temporally complete set of values, involves significant participant burden and is likely to impact consumption behavior [6]. As a consequence, in many studies, alcohol consumption data are sampled at various times throughout pregnancy [7]. However, even when data for the specific time-points are complete, there is frequently missing information about intake during the intervals between study visits. Addressing this missing data problem is critical when the exposure metrics of interest are both timing and amount during pregnancy [8].

The impact of missing data on the validity of estimates largely depends on the reasons data is missing [9]. For example, pregnant women of low socioeconomic (SES) background are more likely to access antenatal care late in pregnancy, enroll late in research studies, and, therefore, have more missing data early in pregnancy [10]. This is problematic as SES is an important determinant of drinking behavior during pregnancy [11]. In addition, women often modify their consumption behavior following pregnancy recognition, which happens at varying times during the first months of pregnancy. While some women stop or reduce drinking immediately upon pregnancy recognition, some heavy drinkers continue to binge in the first trimester or continue heavy drinking throughout the pregnancy [8]. The accuracy of measures irrespective of the presence of missing data, such as the number of drinks consumed only on drinking days, may also provide biased overall estimates depending on when participants are interviewed. Therefore, new approaches for managing the missing data problem are needed.

**FIGURE 1:**
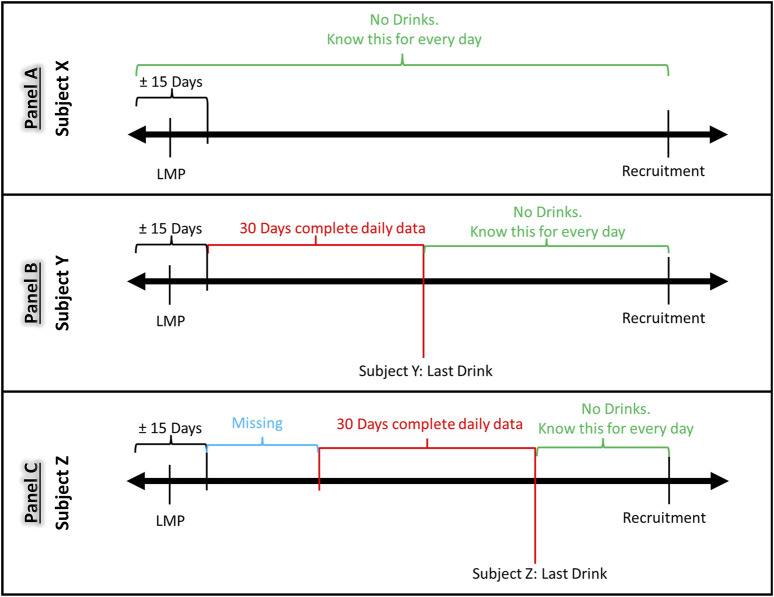
Timing of alcohol consumption during pregnancy and its relation to missing data. Panel **(A)**: Participants who did not drink, or whose last drinking day was prior to their LMP had no missing data. Panel **(B)** Participants who drank but quit drinking within 30 days of the last collection period, had no or less missing data. Panel **(C)**: Participants who reported drinking information 30 days closest to the interview date, had missing information prior to the 30-day period of reported drinking.

The Safe Passage Study conducted by the Prenatal Alcohol and SIDS and Stillbirth Network (PASS) was a prospective investigation of effects of alcohol exposure on multiple fetal and infant outcomes in Cape Town, South Africa and the Northern Plains, USA [12]. In this study, alcohol data were collected using a modification of the Timeline Followback Method (TLFB) [13], in which pregnant women reported drinking data on their last known drinking day and then, for the 30 days prior. While this method was deemed the best self-report system available, the approach, by design, generates a variable amount of missing data per participant. As an example, recent drinkers were more likely to have higher number of missing data points (Figure 1). Because drinking behavior during pregnancy vary by the timing of the pregnancy, as well as day of the week, and most participants had some daily drinking data missing, imputation methods such as last value carry forward and mean imputation were not applicable.

In this paper we describe a method to impute the drinking values on missing days using a machine learning algorithm called k-nearest neighbor (*k-NN*). *k-NN* imputes missing values using pattern recognition without any distributional assumption about the underlying data [14]. The *K-NN* algorithm is particularly suitable for imputation of prenatal drinking data as drinking during pregnancy follows specific patterns depending on pre-pregnancy drinking practices and the length of pregnancy [15, 16]. The *k-NN* algorithm has been used in imputation of missing data in several research areas in the healthcare field including genetics and metabolomics studies [17, 18]. In this paper, we provide the methodological details of the specific application of the *k-NN* algorithm for imputation of PASS exposure data and the validation of these results.

## Methods

### The safe passage study

The Safe Passage Study was a prospective study of a cohort of pregnant women and their infants evaluating the role of prenatal alcohol exposure on incidence of adverse pregnancy outcomes including stillbirth, sudden infant death syndrome (SIDS), and fetal alcohol spectrum disorders (FASDs) of the surviving children. Between August 2007 and January 2015, 11,892 pregnant women (11,083 included here) were enrolled from antenatal clinics in Northern Plains, USA and Cape Town, South Africa. Women were eligible to participate in the study if they were pregnant with one or two fetuses, aged 16 years or older, were at gestational age 6 weeks or later at recruitment and spoke English or Afrikaans. Women were followed throughout the pregnancy and 1 year postnatally. Data on socio-demographic factors, obstetric history, periconceptional drinking and smoking were collected at the enrollment interview. Information on subsequent drinking during pregnancy was updated in study visits following enrollment.

### Ethics

Ethical approval was obtained for each participating PASS network site from their institutional review boards including Stellenbosch University, Sanford Health, the Indian Health Service and from participating Tribal Nations. Written informed consent was obtained from all participants. All data collection and analyses were performed in accordance with the guidelines of the participating institution’s ethical review boards. The research was also overseen by the PASS Network Steering Committee as well as an external Advisory and Safety Monitoring Board.

### Alcohol data collection method and missing data

Alcohol exposure data were collected using a modified validated TLFB [13], which required participants to report details of their drinking on each day for ±15 days from the last menstrual period (LMP) and, at each study visit, the 30 days prior to the last known drinking day. Data were collected on the types and number of drinks, size of the containers, amount of ice in the drink, how many people shared drink, and duration of the drinking episodes [13]. These data were then used to estimate the total amount of alcohol consumed and number of standard drinks on each reported drinking day [19]. Data on drinking were collected during 1–4 prenatal study visits and 1 visit postpartum.

Due to the nature of the modified TLFB data collection design, the number of days with missing data varied by participant as a function of the time of enrollment and number of subsequent visits. The number of days with missing drinking information also varied for each participant depending on the recentness of their drinking. Figure 1 shows examples of how such variation emerged during the period between LMP and the recruitment visit depending on when the last drinking day occurred. Participants who did not drink, or whose last drinking day was prior to their LMP had no missing data (Figure 1A). Participants who drank but quit drinking within 30 days of the last collection period, had less or no missing data (Figure 1B). Participants who continued to drink, and who reported drinking information 30 days closest to the interview date, had missing information prior to the 30-day period of reported drinking (Figure 1C). In this example, if Subject Z drank often, and possibly at a higher volume, she would have a greater number of missing days than women who drink less often. Thus, a summation of drinks over the days will reflect less than the actual consumption and analysis using this exposure metric will be biased.

Let’s assume that subjects X, Y, and Z were enrolled at the same gestational ages for their respective pregnancies. The alcohol consumption of Subject X is depicted in Panel A. Participant X is a non-drinker given no alcohol consumption is reported in the time interval which spans from LMP and recrutiment. Both Subject Y (Panel B) and Subjects Z (Panel C) did report at least an event of alcohol consumption in the same interval. Neverthless, the timing of alcohol intake is different for the participants, thus resulting is the absence (Subject Y) and presence (Subject Z) of data missingess. Considering Subject Y, the time interval between last alcohol intake and LMP is less or equal 30 days, thus there is no gap in alcohol consumption information, resulting in a complete timeline from recruitment back to LMP. On the contrary, Subject Z reported her last drinking event more recently with respect to Subject Y, thus the interval between last alcohol consumption and LMP is greater than 30 days. In this latter case, we have data missing by design of the assessment instrument.

### The k-NN algorithm

**FIGURE 2:**
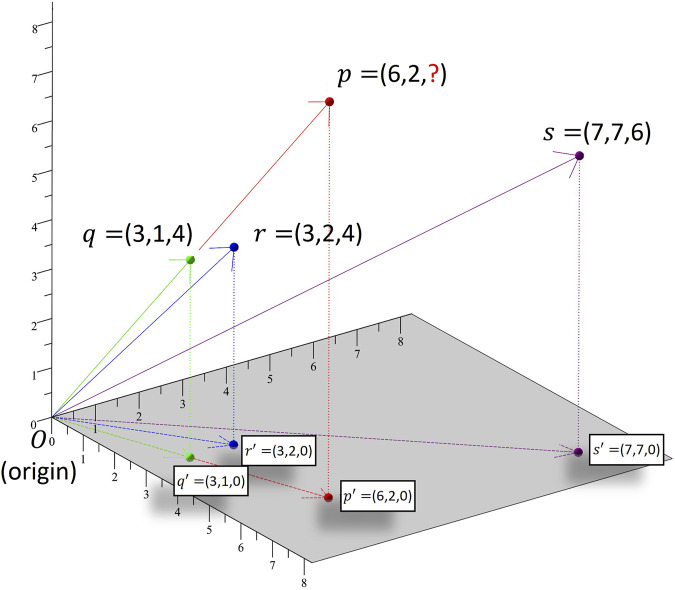
Hypothetical example showing application of k-NN algorithm on drinking data from three subects. Subjects *p*, *q*, *r* and *s* are mapped to points *p*′, *q*′, *r*′ and *s*′, respectively, in 2-dimensional space based on the 2 days for which data are available for all of them. If a subject had *x* drinks on day one and *y* drinks on day two then it is mapped to point (*x*, *y*, 0) on the 2-dimensional *xy* plane.

*k-NN* is a non-parametric machine learning algorithm which can be utilized to impute missing drinking information of a subject based on the information provided by other observations in a given database. Figure 2 displays the imputation of missing data for subject *p* based on the drinking information of subjects with drinking patterns most similar to that of *p*. Similarity in the drinking patterns of two subjects is measured using their *cosine distance*. In this hypothetical example, there are three subjects (*q*, *r* and *s)* for whom estimates of alcohol consumption were collected on three different days during pregnancy. For subject *p* information is missing for the third day. The nearest neighbor for subject p is subject q. The angle between *p*′*O* and *q*′*O* is zero which means that *p* and *q* have exactly the same drinking pattern, as they both consumed three times more drinks on day 1 than on day 2. The next nearest neighbor for subject p is subject r as the angle between them is small. In practice, it is computationally complex to calculate an angle and we can use the *cosine* as a good approximation. Once the *k* nearest neighbors of *p* are identified*,* the weighted average of the drinking data of these neighbors for the day for which *p*’s drinking data are missing is taken as the best estimate of the missing data. The weighted average is taken to assure that the neighbors nearer to *p* have more influence on the predicted value than the ones further away from it. We also scaled the imputed values to individual consumption level. In this example, the scaling adjustment is needed because though *p* and *r* have similar drinking patterns, p is heavier drinker than r. Details of the computation of cosine similarity and scaling adjustment are described in Supplementary Appendix S1.

### Data preparation

We first converted the data to a single record (row) per person, where drinking values were separate variables (columns), one variable for each drinking day starting from day −15 (2 weeks prior to LMP) and ending at day 310 (maximum possible pregnancy length). We used the distance between a fixed date before the start of the study (Saturday, 1 January 2000), and the beginning of pregnancy (i.e., day −15) to find the day of the week the pregnancy started. This was then used to temporally align each subject prior to computation of the cosine distances. For example, when computing the nearest neighbors of a participant *p* whose pregnancy started on a Wednesday, if we encountered another participant *q* whose pregnancy started on a Monday, we aligned day −15 of *p* (a Wednesday) with day −13 of *q* (another Wednesday) and ignored the first 2 days (days −15 and −14) of *q* and the last 2 days (days 309 and 310) of *p.* The rationale behind this alignment is that the drinking behavior often varies by the day of the week [20]. We also Winsorized (capped) the outlier drinking values at 3 SD (21 for South Africa and 28 for Northern Plains sites) to reduce the impact of outlier values in determining the imputed values. As the pattern of drinking in subjects with data missing for a large number of days in pregnancy cannot be established, we excluded subjects who did not have any data in the first trimester and those who were missing more than 200 days of data. Those missing more than 200 days of data were missing data from more than two trimesters and the periconceptional period. The final data set for imputation included 11083 subjects.

#### Assessment of performance

We validated our approach by comparing the actual values from a subset of subjects with no missing data in trimester 1 and the resulting imputed values obtained after random deletion of data for 5 to 15 consecutive days. The first trimester was selected for validation because the proportion of women drinking and the magnitude of their drinking is highest in trimester 1, particularly for the days before pregnancy recognition.

To identify the optimum number of neighbors to be included, we examined the root mean squared error (RMSE) for the predicted drinking values in the deleted segments (Figure 4A) as follows,

$$RMSE=\sqrt{\frac{\sum_{i=1}^{n}{\left({\hat{y}}_{i}-y_{i}\right)}^{2}}{n}}$$

where n is the length of a segment and for 1≤i≤n , $y_{i}$ and $\hat{y_{i}}$ are the actual and predicted value, respectively, of the $i^{th}$ entry of each segment.

We calculated the overall number of correctly imputed segments of drinking status as proportion of accurate classification and plotted it in a confusion matrix (Figure 4B). We ran 500 iterations to estimate the imputation accuracy for the chosen number of neighbors (k = 5). We then calculated absolute differences between actual and predicted values and their confidence interval, for drinking and non-drinking days separately (Supplementary Figure S1).

## Results

### Description of missing data

Participants contributed a total of 3.2 million person-days of observation in the study, of which 0.36 million (11.4%) person-days were missing. Based on the data collected using the TLFB method about 45% of the participants (n = 4,988) had alcohol use data for every single day of their pregnancy while the remaining 55% (n = 6,096) had at least 1 day of alcohol-use data missing. Among the study participants 62% (n = 6,872) were drinkers, i.e., consumed at least 1 drink during pregnancy. Overall, Northern Plains sites had fewer missing data, with over 50% of the participants having 30 or fewer days of missing data (Supplementary Figure S1). Most of the missing data in the South Africa site are from the early trimesters which largely reflects later enrollment at that site, whereas the majority of missing data in the Northern Plains site are in the 3rd trimester (data not shown). This has important implication for imputation, given majority women reduce or stop drinking after pregnancy identification. Early pregnancy missing data are more likely to represent drinking periods compared to late pregnancy.

### Application of *k-NN*

#### Length of reference segment

**FIGURE 3:**
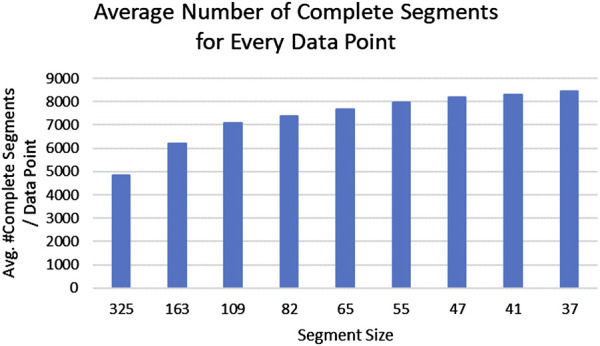
Average number of complete segments without missing data (y-axis) for each segment length (x-axis) are shown. A segment size of approximately 2 months (55 days) retains the majority of the subjects in the reference pool without reducing the ability to identify their drinking patterns.

The largest possible reference segment for each pregnant woman in the PASS data set is 324 days, the maximum length of the pregnancy (310 days) plus 2 weeks before pregnancy. However, as mentioned in a previous section, women with complete data were more likely to be nondrinkers or light drinkers, hence exclusively using them as neighbors would produce an underestimate of true drinking values. We therefore included segments of data without any missing values from all pregnant women as reference data for imputation. The trade-off between selecting a larger or smaller segment size is that smaller segment sizes (e.g., 7 days) allow more segments to be included as reference; but the smaller the segment becomes, the less accurate is the algorithm’s characterization of specific patterns of drinking. We also determined that a reduction of segment sizes below 55 days did not increase available reference segments significantly (Figure 3). A segment size of approximately 2 months (55 days) retained the majority of the subjects in the reference pool without diminishing the ability to identify their drinking patterns.

#### Number of neighbors, k

**FIGURE 4:**
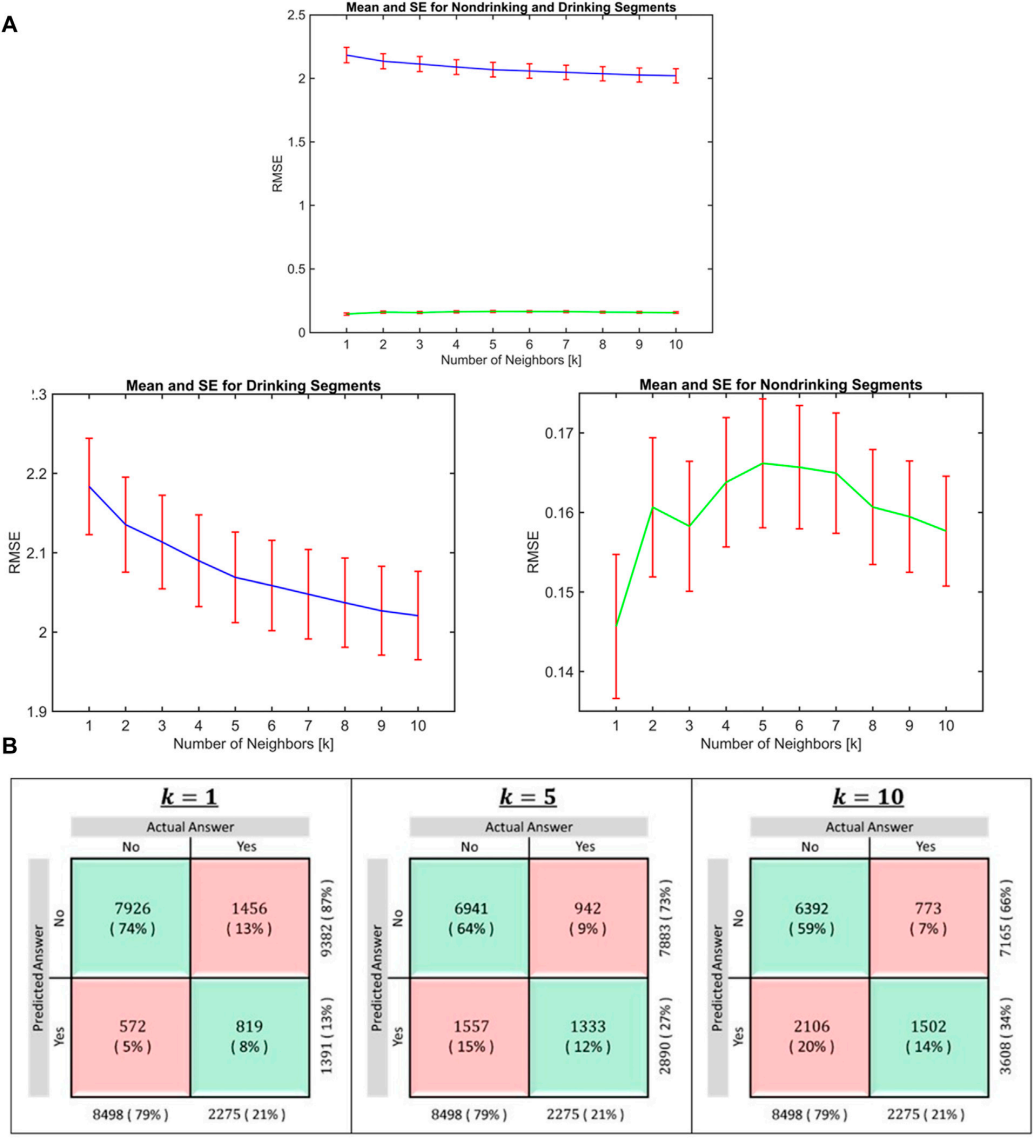
**(A)**: Distribution of RMSE in drinking and nondrinking segments for k = 1 to 10. The mean RMSE value in the drinking segments decreased (blue line) as the value of k is increased and mean RMSE after k = 5 did not decrease substantially. Given the overall RMSE in non-drinking segments (green line) are small, increase in RMSE after inclusion of more than 1 neighbors (K > 1) was very small. **(B)**: Classification accuracy for k = 1, 5, and 10. This confusion matrix shows the proportion of participants classified as drinkers or nondrinkers following imputation after randomly deleting data for 5–15 consecutive days from the first trimester. While the classification accuracy in non-drinking segment is highest when k = 1, the accuracy in drinking segments increased when more than one neighbor is included.

To identify the optimal number of neighbors to be included in imputation, we varied the number of neighbors k from 1 to 10. Figure 4 shows the distribution of root mean square errors (RMSE) for each k for drinking and non-drinking segments separately. For the prediction of nondrinking segments, k = 1 provided the lowest RMSE (panel a) and using k > 1 (multiple neighbors) provided lower RMSE for the prediction of drinking segments. The mean RMSE value in the drinking segments decreased as the value of k is increased, while the increase in RMSE for non-drinking segments after inclusion of more than 1 neighbors (k > 1) was very small, given the mean RMSE in these segments are very small to begin with. We additionally considered the classification accuracy (Figure 4B) and we concluded that k = 5 provided reasonable accuracy for the imputation of both drinking and nondrinking days. The classification accuracy in non-drinking segment is highest when k = 1, while the accuracy in drinking segments increased when more than one neighbor is included. Classification accuracy for non-drinking segments decreased as the number of neighbors increased (Figure 4B). Considering both the RMSE values and classification accuracy in drinking and non-drinking segments, we selected 5 neighbors for the imputation.

#### Imputation accuracy using k = 5

We found the *k-NN* algorithm made exact predictions of drinking status for 76% drinking segments in the site combined analysis. The algorithm predicted nondrinking status (drinking segment or nondrinking segment) accurately in 74% and 58% of the deleted segments in South Africa and Northern Plains respectively (data not shown). We then examined the absolute difference between the actual and predicted values for non-drinking segments (Supplementary Figure S1). Using K = 5, the algorithm predicted nondrinking segments within +/− 1 drinks, for 78.6% of deleted segments in South Africa and 67.6% in the Northern Plains. Notably, in segments where the prediction was not exact, the difference between the predicted and actual values was minimal in terms of the numbers of drinks per drinking segment.

### Average drinking after imputation

Supplementary Figure S2 shows the mean number of drinks per person by trimester before and after imputation. Following imputation, the mean number of drinks in South Africa increased by an average of 2 drinks in first trimester, while the increase for the Northern Plains sites was just below 1 drink in first trimester. Following imputation, the magnitude of increase in mean drinks in South Africa was higher than that in Northern Plains. The Northern Plains had fewer missing data than the South African site. In addition, the proportion of drinkers and drinking volume was lower in the Northern Plains site. Consequently, although many individual drinking values were changed, imputation had a small effect on the average drinking values in Northern Plains sites.

## Discussion

The objective of this article is to illustrate the application of a machine learning algorithm to impute missing daily alcohol consumption data in a prospective study among pregnant women. When pregnant women were asked about alcohol consumption during their prenatal visits, a variable amount of missing data was generated as a consequence of the Timeline follow back data collection method and there were more missing data among recent drinkers. We implemented an extension of a *k-NN* algorithm which accounted for the absence of a “typical/classic” reference group, i.e., training data set with no missing days. To our knowledge, the present report is the first to describe this method to impute missing alcohol consumption data in a longitudinal study among pregnant women. Validation of our approach showed high agreement between actual and predicted drinking values.

There is a paucity of studies addressing the potential bias introduced by missing data as well as a lack of methodological tool to impute missing data in alcohol and drug use research [21]. Published work has not yet reported the performance of any machine learning method for imputation of missing alcohol data. In a simulated dataset, Hallgren et al. compared methods of imputation including complete case analysis, last observation carried forward, the worst-case scenario of missing equals any drinking or heavy drinking, multiple imputation (MI), full information maximum likelihood (FIML) and concluded that MI and FIML yielded less biased estimates [22, 23]. A recent study by Grittner et al. also found MI produced least bias based on their work in a longitudinal study in Denmark with five alcohol measurements over a period of 5 years [24]. However, all methods in the study including the MI produced an underestimate of the actual drinking level. In addition, MI models are originally recommended for imputation of a single value per subject [25]. To impute irregularly spaced missing longitudinal data as in PASS, complex extensions of MI would be needed [26].

There are several advantages with using a non-parametric algorithm such as the *k-NN* algorithm for imputation of missing data. The majority of standard software packages rely on the assumption of normal distribution of multivariate data, therefore imputation of repeated longitudinal data in most software options is challenging [26]. In the PASS dataset, alcohol data were collected at the daily level resulting in a high total volume of both data per participant and associated missing data. Alcohol consumption in pregnancy is highly skewed with the majority of the drinking concentrated in the first trimester. We observed similar pattern in our data that there was also a gradually decreasing drinking pattern among many study subjects. In such scenarios, a nonparametric method such as *k-NN* has the advantage of not making a distributional assumption.

The sample size required to achieve a reliable performance of k*-NN* imputation depends on the variability of the data being imputed. Specifically, the higher the variability in the sample, the greater the number of observations needed to derive making inference from that data. The choice of number of neighbors (k) depends on the nature of the problem under investigation, the available data as well as downstream analyses goals. On average, a higher number of neighbors results in a greater prediction accuracy but presents the limitation of standard deviations to be significantly inflated [27]. In most scenarios, the use of a smaller k is a good compromise between performance and preservation of original distribution of the data. In fact, higher number of neighbors fails the purpose of detecting the most appropriate observations like the one under consideration. In addition, computational load in terms of neighbor searching and storing the training set must be taken into consideration [28]. While it is not possible to provide *a priori* indication on the optimal number of neighbors for a given dataset without conducting a sensitivity analysis, in the context of our work we found k = 5 as a reasonable trade-off between RMSE for drinking and non-drinking segments. Similar values of k were also reported in prior studies [29, 30].

**FIGURE 5:**
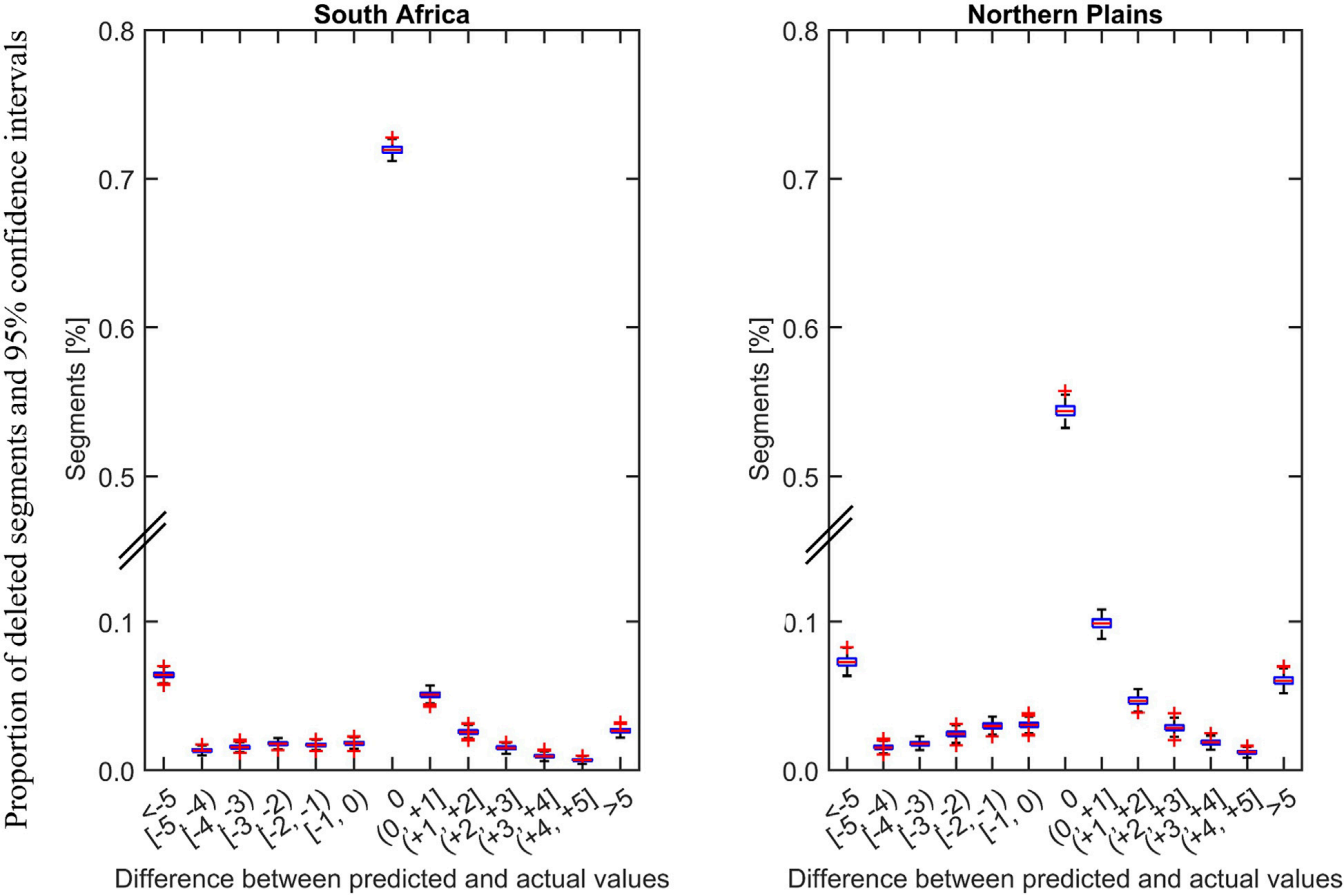
Distribution of difference between predicted and actual values by study site are shown. The differences and their 95% confidence intervals were obtained from 500 iterations to by deleting random segments of non-missing data of 5–15 days length from first trimester. The proportion of exact prediction (difference = 0) is higher for South African data compared to data from the US. Notably, the in segments where the prediction was not exact the difference between the predicted and actual values were minimal in terms of the numbers of drinks per drinking segment.

To evaluate imputation performance, we used a confusion matrix showing the imputation accuracy in the binary drinker and non-drinker classification (Figure 4: Panel b) and mean absolute difference between the predicted and actual daily drinking values with their confidence intervals (Figure 5). Our choice of imputation metric is dictated by the type of data to be imputed and our downstream analysis goal. The imputed data we derived was used for such cluster analyses identify distinct group of participants with similar drinking patterns considering the timing and quantity of the drinks consumed [31]. While our validation data shows that k-NN can impute missing drinking data with high accuracy, the algorithm cannot overcome bias introduced in the data when participants report drinking days as non-drinking days.

The *k-NN* algorithm is increasingly used to impute missing data in research with high volume data such as genetics and metabolomics studies [32, 33]. In several recent reports the *k-NN* algorithm was shown to produce the smallest imputation error compared to methods such as mean and median imputation, Bayesian linear regression, K-Means, K-Medoids clustering algorithms [34, 35]. However, some studies reported that simpler methods such as mean or median replacement were as adequate as methods like *k-NN* when imputation was followed by clustering of genetic data [36]. On the other hand, some have reported slightly better performance of random forest over *k-NN* to impute metabolomics data [37]. Another study noted improvement of performance of *k-NN* when additional information such as SES and demographic data were included in the prediction model [38]. We have used cosine distance to measure the similarity in the drinking patterns of two subjects. Chomboon et al evaluated 11 distance measures which showed that several other distance measures perform adequately [39]. Future studies could evaluate performance of multiple distance measures in imputing alcohol data. The validity and accuracy of imputation will likely vary with the data type, data structure, mechanism of missingness, amount of missing data and the choice of downstream analyses. Therefore, future studies are needed to evaluate the performance of different machine learning algorithms to impute alcohol consumption data.

In this paper, we provide a comprehensive description of imputation of prenatal alcohol data using *k-NN* algorithm with high accuracy. Data collection methods like Timeline follow back [40] and the food frequency questionnaires [41] collect extensive longitudinal consumption data but they are prone to informative missing data. The methodologic details presented in this paper are of high relevance to various research areas including substance use and nutrition research that suffer from missing data in longitudinal studies.

### Short summary

Missing data are a source of bias in many epidemiologic studies. This is problematic in alcohol research, where data missingness is linked to drinking behavior. The Timeline Followback Method (TLFB) for assessment of alcohol consumption, where participants report drinking on their last known drinking day and for the 30 days prior, is deemed the best self-report system. However, TLFB method, by design, generates a variable amount of missing data per participant. In this paper, we describe a method to impute the drinking values on missing days using a machine learning algorithm called k-nearest neighbor (k-NN). k-NN imputes missing values using pattern recognition without any distributional assumption about the underlying data. This algorithm is particularly suitable for imputation of prenatal drinking data as drinking during pregnancy follows specific patterns depending on pre-pregnancy drinking status and the length of pregnancy. The k-NN algorithm has been used in the imputation of missing data in several research areas in the healthcare field, including genetics and metabolomics studies. Using data from a prospective cohort study among 11083 pregnant women from the United States and South Africa, we demonstrate that the k-NN algorithm can be used to impute missing alcohol data during pregnancy with high accuracy.

## Data Availability

The data analyzed in this study is subject to the following licenses/restrictions: The data may be made available up on request to be submitted to the corresponding author. Requests to access these datasets should be directed to as4823@cumc.columbia.edu.

## Supplementary material

Supplementary Figure 1 The distribution of missing data by study site is shown. Northern Plains sites had fewer missing data, with over 50% of the participants having 30 or fewer days of missing data.

Supplementary Figure 2 Average drinks per trimester before and after imputation, by study sites and combined. Following imputation, the mean number of drinks in South Africa increased by an average of 2 drinks in first trimester, while the increase for the Northern Plains sites was just below 1 drink in first trimester.

## Abbreviations

*k-NN*, K Nearest Neighbor; LMP, Last menstrual period; MI, multiple imputation; PAE, Prenatal alcohol exposure; PASS, Prenatal Alcohol and SIDS and Stillbirth Network; SES, Socioeconomic status; SIDS, sudden infant death syndrome; TLFB, Timeline Followback Method; FIML, full information maximum likelihood; RMSE, Root mean square errors.
