# Supplementary figures and images for: K-nearest neighbor algorithm for imputing missing longitudinal prenatal alcohol data
Source: Adv Drug Alcohol Res. 2025 Jan 28;4:13449. doi: 10.3389/adar.2024.13449 (PMC11811783; doi:10.3389/adar.2024.13449)

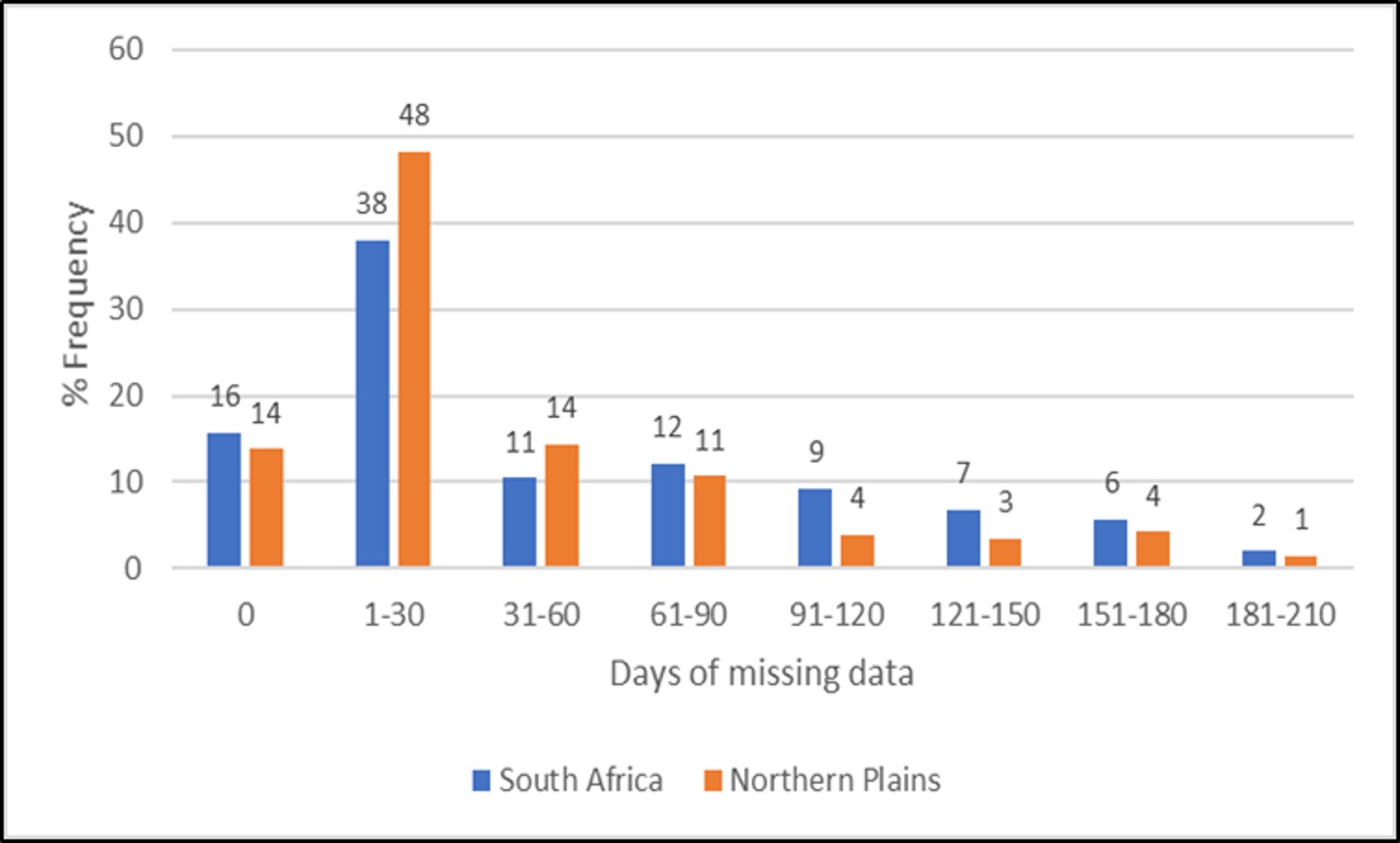

Supplement: Supplementary file 1 [file Image1.JPEG]

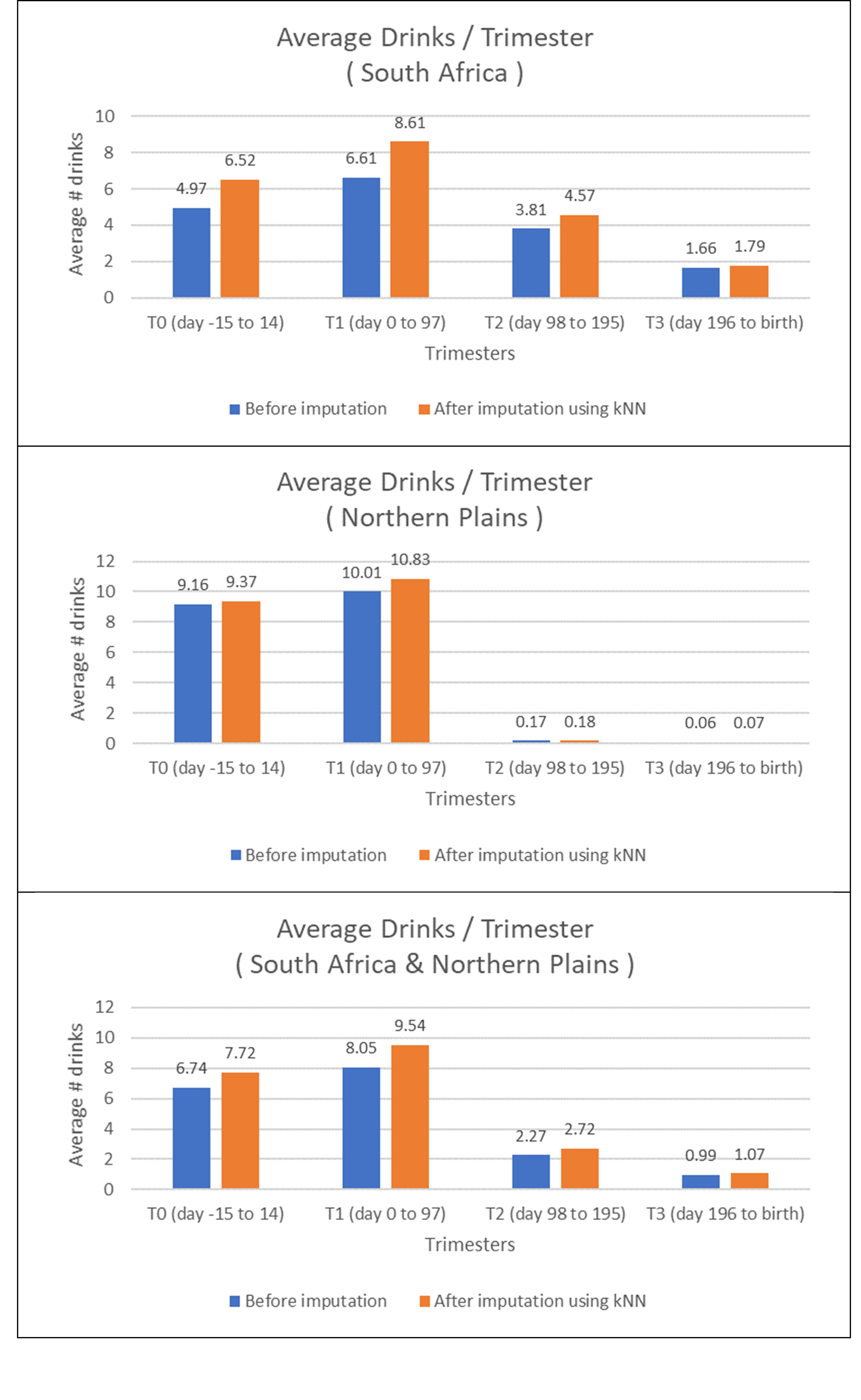

Supplement: Supplementary file 2 [file Image2.JPEG]
