# Supplementary material for: K-nearest neighbor algorithm for imputing missing longitudinal prenatal alcohol data
Source: Adv Drug Alcohol Res. 2025 Jan 28;4:13449. doi: 10.3389/adar.2024.13449 (PMC11811783; doi:10.3389/adar.2024.13449)
Supplement: Supplementary file 3 [file DataSheet1.docx]

**Appendix 1: Details of the kNN using hypothetical example**

In this hypothetical example there are three subjects *q*, *r* and *s* for which estimates of alcohol consumption were collected (e.g., number of drinks per day) on three different days (d) during pregnancy. However, for another subject (*p)* information is missing for the third day. Figure 2 shows this example. As a first step, data from each of these four subjects, can be mapped to *d* - 1 dimensional space using data for, in this case, the two days for which all four of them have data. In Figure 2, subjects *p*, *q*, *r* and *s* are mapped to the two-dimensional space for which all four subjects have data and are labeled points *p*′, *q*′, *r*′ and *s*′, respectively. In this example, since subject *r* had 3 drinks on day one and 2 drinks on day two, it is mapped to point (3, 2) in 2-dimensional space (shown as point (3, 2, 0) in the figure). To find the degree of similarity between the drinking habits of subject *p* and another subject, e.g. subject *r*, we connect *p*′ and *r*′ to the origin *O* and measure the angle between *p*′*O* and *r*′*O*. The smaller the angle, the greater the similarity. In Figure 2, the angle between *p*′*O* and *q*′*O* is zero which means that *p* and *q* have exactly the same drinking pattern, in that both consumed three times more drinks on day 1 than on day 2. The angle between *p*′*O* and *r*′*O* is larger than that between *p*′*O* and *q*′*O* which means that *r* is less similar to *p* than *q* is to *p*. On the other hand, the angle between *p*′*O* and *r*′*O* is smaller than that between *p*′*O* and *s*′*O* meaning that *r* is more similar to *p* than *s* is to *p*. Based on these angular distances the algorithm defines the nearest neighbors of subject *p*. For any given value of k (> 0), the *k nearest neighbors* of *p* are those *k* subjects in the dataset that form the *k* smallest set of angles with *p*. Those are the *k* subjects that have drinking patterns most similar to that of *p* and are most useful in imputing its missing data values. However, in practice, it is computationally complex to calculate an angle and we can use the *cosine* as a good approximation. The *cosine* of an angle is computed as follows. If both *p* and *r* have drinking data for *h* different days given by $\langle p_{1},p_{2},\ldots,p_{h}\rangle$ and $\left\langle r_{1},r_{2},\ldots,r_{h} \right\rangle$, respectively, where 1 ≤ *h* ≤ *d*, then the cosine distance between them is given by the following expression.

$$\cos\left( \left\langle p_{1},p_{2},\ldots,p_{h} \right\rangle,\left\langle r_{1},r_{2},\ldots,r_{h} \right\rangle\right)=\frac{\sum_{i=1}^{h} p_{i}r_{i}}{\sqrt{\left( \sum_{i=1}^{h} p_{i}^{2} \right)\left( \sum_{i=1}^{h} r_{i}^{2} \right)}}$$

The smaller the angle between two subjects, the larger the cosine distance between them provided all angles lie between $0^{\circ}$ and $90^{\circ}$ which is always true in our case because drinking data (regardless the employed estimator) are lower bounded to zero (i.e., number of drinks per day). That means, the *k* nearest neighbors of *p* are those *k* subjects in the data set that have the *k* largest cosine distances from *p*.

Once the *k* nearest neighbors of *p* are identified*,* the weighted average of the drinking data of these neighbors for the day for which *p*’s drinking data are missing is taken as the best estimate of the missing data. The obtained average represents k-NN’s prediction of the number of drinks subject *p* had on the h-ith missing. The weight given to each neighbor, e.g. *r*, is of the form $w_{r}=\sigma_{r}\delta_{r}$, where $\sigma_{r}$ is a scaling factor and $\delta_{r}$ is a distance factor. We set $\sigma_{r}={|p'O|}/{|r'O|}$, where $|p'O|$ and $|r'O|$ represent the *Euclidean distance* (i.e., the straight-line distance) of *p*′ and *r*′, respectively, from the origin. The scaling adjustment is needed because though *p* and *r* may have similar drinking patterns, one may be a heavier/lighter drinker than the other. For example, in Figure 2, *p* and *q* have the same drinking pattern, but *p* is still a heavier drinker (precisely, twice as heavy) than *q*. We set $\delta_{r}$ to the square of the cosine distance between *p* and *r*. This distance factor assures that the neighbors nearer to *p* have more influence on the predicted value than the ones further away from it.
